# The Repeatable Battery for the Assessment of Neuropsychological Status for Hearing Impaired Individuals (RBANS-H) before and after Cochlear Implantation: A Protocol for a Prospective, Longitudinal Cohort Study

**DOI:** 10.3389/fnins.2016.00512

**Published:** 2016-11-15

**Authors:** Annes J. Claes, Griet Mertens, Annick Gilles, Anouk Hofkens-Van den Brandt, Erik Fransen, Vincent Van Rompaey, Paul Van de Heyning

**Affiliations:** ^1^University Department of Otorhinolaryngology, Head and Neck Surgery, Antwerp University HospitalAntwerp, Belgium; ^2^Faculty of Medicine and Health Sciences, University of AntwerpAntwerp, Belgium; ^3^Department of Human and Social Welfare, University College GhentGhent, Belgium; ^4^StatUa Center for Statistics, University of AntwerpAntwerp, Belgium

**Keywords:** RBANS-H, cochlear implantation, cognition, older adults, RBANS

## Abstract

**Background:** Currently, an independent relationship between hearing loss and cognitive decline in older adults is suggested by large prospective studies. In general, cochlear implants improve hearing and the quality of life in severely to profoundly hearing impaired older persons. However, little is known about the effects of cochlear implantation on the cognitive evolution in this population.

**Aim of the study:** The primary goal of this prospective, longitudinal cohort study is to explore the cognitive profile of severely to profoundly postlingually hearing impaired subjects before and after cochlear implantation. In addition, the current study aims to investigate the relationship between the cognitive function, audiometric performances, quality of life, and self-reliance in these patients.

**Methods:** Twenty-five patients aged 55 or older, scheduled for cochlear implantation, will be enrolled in the study. They will be examined prior to implantation, at 6 and 12 months after implantation and annually thereafter. The test battery consists of (1) a cognitive examination, using the Repeatable Battery for the Assessment of Neuropsychological Status adapted for Hearing impaired persons (RBANS-H), (2) an audiological examination, including unaided and aided pure tone audiometry, speech audiometry in quiet and speech audiometry in noise, (3) the administration of four questionnaires evaluating quality of life and subjective hearing benefit and (4) a semi-structured interview about the self-reliance of the participant.

**Discussion:** Up until now only one study has been conducted on this topic, focusing on the short-term effects of cochlear implantation on cognition in older adults. The present study is the first study to apply a comprehensive neuropsychological assessment adapted for severely to profoundly hearing impaired subjects in order to investigate the cognitive capabilities before and after cochlear implantation.

**Trial registration:** The present protocol is retrospectively registered at Clinical Trials (ClinicalTrials.gov) on June 9th, 2016. The first participant was enrolled on June 22nd, 2015. The protocol identifier is NCT02794350.

## Background

Over the past decade the relationship between hearing loss and cognitive decline in the older population has gained more research interest. Large prospective studies have found an independent relationship between hearing loss on the one hand and age-related cognitive decline and incident dementia on the other hand (Lin et al., 2011, 2013; Gallacher et al., 2012; Gurgel et al., 2014). The basis of this association remains unclear. For instance, hearing loss may accelerate cognitive decline in older adults and therefore may be a risk factor of this pathology. Alternatively, hearing loss could be an early symptom of cognitive decline and could be an effect rather than a cause of the cognitive impairment. A common cause that influences both pathologies may be a third underlying mechanism of the association between hearing loss and cognitive decline (Martini et al., 2014; Peracino, 2014). As hearing aids can improve the hearing and contribute to reestablishing the individual's participation in society, they could have a positive effect on the expected trajectory of cognition. However, the results of studies investigating the effect of hearing aids on cognitive function in older adults are variable (Allen et al., 2003; van Hooren et al., 2009; Acar et al., 2011; Magalhaes and Iorio, 2011; Kalluri and Humes, 2012; Amieva et al., 2015; Dawes et al., 2015; Meister et al., 2015). Moreover, in case of a positive relationship between hearing aid use and cognition, the direction of causality is not clear. Rather than hearing aid use improving cognitive abilities, individuals who are cognitively well-functioning, may tend to seek and obtain hearing aids more often (Dawes et al., 2015). Very limited research has been conducted concerning the impact of cochlear implantation on the cognitive capabilities of older adults. Miller et al. (2015) provided an overview of existing literature on the question as to whether cochlear implantation in older adults with a severe to profound hearing loss modifies the expected evolution of cognitive decline. Based on the reviewed articles, the authors concluded that there is a lack of well-designed prospective studies evaluating changes in cognition after cochlear implantation. Recently, Mosnier et al. (2015) published a multi-center, prospective, longitudinal study concerning the impact of cochlear implantation on the cognitive capabilities in 94 adults aged 65–85 years. The investigators established improvements in preoperatively impaired cognitive capabilities at 6 months and 1 year after implantation.

The first goal of this longitudinal cohort study is to explore prospectively the cognitive profile of severely to profoundly hearing-impaired patients aged 55 or more, before and after cochlear implantation, both in the short term and the long term. Secondly, the aim is to investigate the relationship between the cognitive function, audiometric performances, quality of life and self-reliance in these patients.

## Methods

### Study protocol

This is a single-center, currently ongoing study, conducted at the Rehabilitation Center for Communication Disorders of the Department of Otorhinolaryngology of the Antwerp University Hospital, Belgium. Subjects are invited to participate in the study if they meet the following inclusion criteria: (1) The subject meets the Belgian national criteria for reimbursement of a cochlear implant, (2) the subject suffers from a postlingual hearing impairment, (3) the subject is scheduled for a first cochlear implantation and (4) the subject is aged 55 or older. The national criteria for reimbursement of a cochlear implant are (A) bilateral severe to profound hearing loss (pure tone average at 500, 1000, and 2000 Hz equal to or exceeding 85 dB HL), (B) brainstem evoked response audiometry results in a threshold of peak V at 90 dB nHL or more and (C) speech recognition scores of 30% or less for Dutch open-set monosyllabic words presented at 70 dB SPL in quiet in the best aided condition. Subjects who do not meet the national criteria for reimbursement and pay the implantation themselves are not included in the study. The case is multidisciplinary evaluated, all contra-indications for cochlear implantation are to be taken into account and the expectations of the person toward the rehabilitation process and the outcomes are thoroughly discussed by the clinician to remain realistic.

The participants are evaluated by an experienced, Good Clinical Practice (GCP) certificated audiologist (Master of Science) prior to implantation and subsequently 6 months and 12 months after activation of the speech processor and annually from then on (Table 1). The test battery consists of a cognitive assessment to evaluate five different cognitive domains, an audiological examination, four questionnaires to assess the impact of the hearing disability and the quality of life (QoL), and a semi-structured interview in which the self-reliance of the patient is discussed.

**Table 1:** **Time schedule of assessments and intervention**.

	**Preoperatively**	**Cochlear implantation**	**6 months** after first fitting	**12 months** after first fitting	**yearly**
	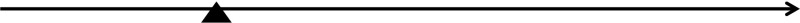				
Cognition	RBANS-H		RBANS-H	RBANS-H	RBANS-H
MOM	Tone audiometry		Tone audiometry	Tone audiometry	Tone audiometry
	- unaided (inserts)		- unaided (inserts)	- unaided (inserts)	- unaided (inserts)
	- aided (free field)		- aided (free field)	- aided (free field)	- aided (free field)
	Speech audiometry in quiet (NVA)		Speech audiometry in quiet (NVA)	Speech audiometry in quiet (NVA)	Speech audiometry in quiet (NVA)
	- unaided (headphones)		- aided (free field)	- aided (free field)	- aided (free field)
	- aided (free field)				
	Speech audiometry in noise (LIST)		Speech audiometry in noise (LIST)	Speech audiometry in noise (LIST)	Speech audiometry in noise (LIST)
	- unaided (headphones)		- aided (free field)	- aided (free field)	- aided (free field)
	- aided (free field)				
	HISQUI_19_		HISQUI_19_	HISQUI_19_	HISQUI_19_
Subjective benefit, Self-reliance, CI usage	Questionnaires		Questionnaires	Questionnaires	Questionnaires
	- NCIQ		- NCIQ	- NCIQ	- NCIQ
	- SSQ12		- SSQ12	- SSQ12	- SSQ12
	- HADS		- HADS	- HADS	- HADS
	Interview		Interview	Interview	Interview

### Primary outcome measurement: RBANS-H

The primary outcome measurement is the change in cognitive capabilities following implantation, as measured by means of the Repeatable Battery for the Assessment of Neuropsychological Status (RBANS, Randolph et al., 1998), adjusted to test Hearing impaired subjects (RBANS-H). The RBANS assesses five cognitive domains, i.e., *Immediate Memory, Visuospatial/constructional, Language, Attention*, and *Delayed Memory* (Table 2). The test consists of 12 subtests and the score on each subtest contributes to one of the five domains. The Dutch version of the original English RBANS is obtained by the process of forward-backward translation. In order to test subjects with a severe hearing impairment a number of adjustments to the standard RBANS had to be made (Phillips, 2016). The administration of the test is accompanied by the use of a PowerPoint presentation shown on an external computer screen connected to a laptop. By means of this presentation all the oral instructions are supported by written explanations to ascertain that the subject understands the instruction even though he or she does not hear what is being said. In addition, the stimuli in the subtests *List Learning, Story Memory, Digit Span*, and *List Recognition* are not only presented auditorily but also visually, making it possible to test the subject in the same reliable way before and after implantation. All the adjustments were made in accordance to the guidelines of the RBANS, as discussed in the manual on page 15 “Testing Examinees With Physical or Language Impairments” (Randolph, 2012). The RBANS was ordered on the website of Pearson Clinical (http://www.pearsonclinical.com).

**Table 2:** **The five cognitive domains and twelve subtests of the RBANS**.

**Immediate memory**	**Visuospatial/ constructional**	**Language**	**Attention**	**Delayed memory**
(1) List Learning	(3) Figure Copy	(5) Picture Naming	(7) Digit Span	(9) List Recall
(2) Story Memory	(4) Line Orientation	(6) Semantic Fluency	(8) Coding	(10) List Recognition
				(11) Story Recall
				(12) Figure Recall

The domain *Immediate Memory* consists of two subtests: *List Learning* and *Story Memory*. In the first subtest, 10 semantically unrelated words are orally and visually presented to the subject. The words are presented on an external screen in lowercase e letters at a 2-s rate. Within the 2-s period the item is visible to the subject for 1.25 s, followed by a 0.75-s interval between the items. The examiner reads the words aloud when they appear on the screen so the subject receives audio-visual information. The subject is asked to repeat as many words as possible after each of four learning trials. In the second subtest, *Story Memory*, a twelve-item short story is presented over two trials. The story is presented visually in three separate parts and read aloud simultaneously at a slow reading speed. After each presentation the subject is asked to recall as much of the story as he or she can remember. A verbatim criterion is used.

The next two subtests, *Figure Copy* and *Line Orientation*, contribute to the second cognitive domain *Visuospatial/constructional*. The first subtest involves copying a geometric figure comprising ten parts, each evaluated for correctness and completeness on the one hand and proper location in relation to the rest of the figure on the other hand. The figure remains visually available while copying. In the second subtest of the *Visuospatial/Constructional* domain, a series of 13 identical lines, radiating out from a single point and spanning 180 degrees, are shown. Below this semi-circular, fan-shaped pattern of numbered lines (1–13), two lines of equal length that match two of the lines from the array are displayed. The subject is asked to give the numbers or point to the two lines that are identical in orientation to the two target lines.

The cognitive domain *Language* includes the subtests *Picture Naming* and *Semantic Fluency*. In the first subtest, 10 line drawings are to be named by the subject. In case of an obvious misperception, a semantic cue is given. The second subtest involves the generation of as many examples as possible from a given semantic category such as fruits and vegetables within 1 min.

The subtest *Digit Span* and the subtest *Coding* contribute to the domain *Attention*. The first of these two subtests is comparable to the subtest *Digits Forward* on the WAIS (Wechsler, 1955). In this test, a string of digits is read aloud and simultaneously shown on the computer screen. Each digit is visible for 1 s with a 0.75-s interval between the digits. The subject is asked to repeat the string in the same order. The length of the string increases by one, from two to nine digits. Two strings are provided for each length, but the second string of the same length is only read if the first one failed. In the subtest *Coding*, a page filled with symbols is presented to the subject and the subject is asked to fill in the number corresponding to each symbol using the key on top of the page. In this key the nine simple symbols are represented horizontally with the corresponding number (1–9) underneath it. The score is calculated as the total number of items correctly completed within 90 s.

The domain *Delayed Memory* comprises four subtests: *List Recall, List Recognition, Story Recall*, and *Figure Recall*. In the *List Recall* subtest, the subject is asked to recall as many words as he or she can remember from the list of 10 words learned previously in the *List Learning* subtest. In the subtest *List Recognition*, 20 words are audio-visually presented to the subject of which 10 words were on the original list (targets) and 10 were not (distractors). The 20 words in this test are transcribed on the screen. The examiner shows the next word at a variable speed depending on the reaction speed of the subject and reads the word aloud when it is shown on the screen. The subject is asked to declare whether each word was on the original list or not. In the *Story Recall* subtest, the subject is asked to recall as many details as he or she can from the story learned in the *Story Memory* subtest. In the last subtest, *Figure Recall*, the subject is asked to draw all the elements of the figure from the *Figure Copy* subtest that he or she can recall without visual display of the figure.

### Secondary outcome measurements

#### Audiological examination

In all subjects an audiological follow-up test is performed which is called the Minimal Outcome Measurements (MOM) (Kleine Punte and Van de Heyning, 2013) (Table 1). This examination includes pure tone audiometry, speech audiometry in quiet, speech audiometry in noise performed in a sound booth and the Hearing Implant Sound Quality Index (HISQUI_19_) (Amann and Anderson, 2014).

##### Unaided and aided hearing thresholds

According to current clinical standards (ISO 8253-1, 2010), unaided pure tone audiometry for air conduction is performed at 125, 250, 500, 1000, 2000, 3000, 4000, 6000, and 8000 Hz using a 2-channel Interacoustics AC-40 audiometer and insert earphones in a sound treated booth. Bone conduction thresholds are tested at 250, 500, 1000, 2000, 3000, and 4000 Hz. The best-aided thresholds with hearing aid(s) and/or cochlear implant (CI) are measured through free field audiometry with warble tones. The loudspeaker is placed at a distance of one meter in front of the subject at ear level. The thresholds are tested at 125, 250, 500, 1000, 2000, 3000, 4000, 6000, and 8000 Hz. If the subject wears two hearing systems, either two hearing aids (preoperatively) or a hearing aid and a CI (postoperatively), the benefit with each hearing system separately is measured as well.

##### Speech audiometry in quiet

Speech reception in quiet is measured using the Dutch open-set NVA lists developed by the Nederlandse Vereniging voor Audiologie (NVA) or Dutch Society for Audiology (Wouters et al., 1994; Bosman et al., 1995). Each list consists of 12 monosyllabic words (consonant-vowel-consonant) of which one is a training item. One list is presented at 65 dB SPL, by means of headphones (unaided, preoperatively) or in free field (aided, pre- and postoperatively) with a loudspeaker at 0° azimuth. The speech recognition score is the percentage of correctly identified phonemes.

##### Speech audiometry in noise

The speech reception in noise is assessed by means of the Leuven Intelligibility Sentences Test (LIST) using an adaptive procedure (van Wieringen and Wouters, 2008). This speech material is developed to quantify the speech understanding in subjects with severely impaired hearing. The frequency spectrum of the noise signal is equal to the long-term average speech spectrum of the sentences. The level of the noise is fixed at 65 dB SPL, while the level of the speech signal is altered depending on the response of the patient. If the subject repeats the keywords of the sentence correctly, the level of the next sentence is decreased by 2 dB SPL. If the subject fails to repeat the keywords, the level is increased by 2 dB SPL. Each list consists of 10 sentences and the speech reception threshold (SRT) is calculated as the mean level of the last five sentences together with the level of the imaginary 11th sentence of the list. This speech in noise test is performed preoperatively in an unaided situation using headphones and both pre- and postoperatively in an aided, free field situation with the loudspeaker in front of the subject at a distance of one meter.

##### HISQUI_19_

The HISQUI_19_ is a 19-item questionnaire to quantify the degree of self-perceived auditory benefit experienced by CI users in everyday communication situations (Amann and Anderson, 2014). The 19 items are rated on a 7-point Likert scale ranging from always (99%) to never (1%). To calculate the overall score the corresponding numerical value of each item (from always = 7 to never = 1) is added. Uncompleted items and the response option “not applicable” correspond to 0 in this calculation. A total score of less than 30, 30–59, 60–89, 90–109, and 110–133 is respectively classified as a very poor, poor, moderate, good, and very good self-perceived auditory benefit. The validated Dutch version of the questionnaire is used (Mertens et al., 2015). The 19 items of this questionnaire are listed and added as Supplementary Material available online.

#### Questionnaires

##### NCIQ

The Nijmegen Cochlear Implant Questionnaire (NCIQ) is a Dutch self-assessment health-related quality of life instrument developed for use in CI users (Hinderink et al., 2000). The questionnaire consists of 60 questions divided into three principal domains: Physical, Psychological, and Social. The first domain comprises three subdomains, namely Basic sound perception, Advanced sound perception, and Speech production. The Psychological domain contains the subdomain Self-esteem and the Social domain handles questions about Activity limitations and Social interactions. Each subdomain covers 10 statements. Each statement is rated on a 5-point Likert scale ranging from “Never” to “Always” (Statement 1–55) or from “No” to “Quite well” (Statement 56–60). A list of the statements of this questionnaire is added as Supplementary Material available online.

##### SSQ12

The SSQ12 (Noble et al., 2013) is a short form of the Speech, Spatial, and Qualities of Hearing scale (Gatehouse and Noble, 2004). It is developed for use in clinical research and rehabilitation settings to measure a range of hearing disabilities across several domains such as speech in noise, speech in speech, localization, distance, and movement, segregation and listening effort. The 12 items of this questionnaire are rated on a visual analog scale from 0 to 10 and the overall score is calculated by taking the average of the scores on these 12 items. The full questionnaire is added as Supplementary Material available online.

##### HADS

To detect states of depression and anxiety the Hospital Anxiety and Depression Scale (HADS) is used (Zigmond and Snaith, 1983). This self-assessment questionnaire consists of seven items in the subscale Depression and seven items in the subscale Anxiety and distinguishes clearly between both emotional disorders. A list of the items of the HADS are available online as Supplementary Material.

#### Interview

In a semi-structured interview, the following topics are covered: Hearing loss, Education and profession, Rehabilitation, Self-reliance and Family. A detailed overview of the different items discussed in the interview is given in Table 3.

**Table 3:** **The five main topics and the different items discussed in the semi-structured interview**.

**Hearing loss**	**Education and profession**	**Rehabilitation**	**Self-reliance**	**Family**
Onset	Years of formal education	Preoperative	Housing	Birth order
Etiology	Degree of studies	Postoperative	Mobility	Number of (grand) children
Device use	Profession		Eating and cooking	Number of siblings
Tinnitus			Personal hygiene	
			House holding	
			Sense of danger	
			Communication	
			Activities	
			Participation in society	
			Mental and fysical health	

### Stepwise procedures

The minimal outcome measurements (MOM), consisting of unaided and aided audiometry, speech audiometry in quiet, speech audiometry in noise and the HISQUI_19_, are part of the standard clinical practice of care and follow-up of CI users in the Antwerp University Hospital. These measurements are performed preoperatively, at three, 6 and 12 months after activation of the speech processor and from then on annually. If the subject agrees to participate in the study, the RBANS-H, the semi-structured interview and the three questionnaires, NCIQ, SSQ12 and HADS, are added to the standard follow-up procedure preoperatively, at 6 and 12 months after activation of the speech processor and annually onwards, without additional appointments.

The three questionnaires are sent by mail to the participants 2 weeks prior to the appointment. The participants are asked to fill out these questionnaires at home and return them at the time of the testing. During the appointment the minimal outcome measurements are performed first, in the previously listed order. The MOM is followed by the cognitive assessment and the semi-structured interview. At the end of the appointment the examiner verifies whether the questionnaires are fully completed by the participant. The complete testing takes approximately one and a half to two hours. In the case of a postoperative appointment, the speech processor is fitted, if needed, prior to the MOM.

### Ethics

This study is carried out in accordance with the recommendations of the ethics committee of the Antwerp Univerity Hospital with written informed consent from all subjects. All subjects give written informed consent in accordance with the Declaration of Helsinki. The protocol was approved by the ethics committee of the Antwerp University Hospital (protocol number 15/17/181) on June 15th, 2015.

### Statistics and anticipated results

The sample size calculation is based on the primary outcome variable, RBANS-H total score. A sample of 19 persons is needed to obtain 80% power using a paired *t*-test to detect a Cohen's d of 0.7 at an alpha level of 0.05. Due to the age of the study population and the duration of the longitudinal study, a 20% dropout rate is taken into account. The targeted total sample size is therefore adjusted to 25.

Descriptive statistics will be used to summarize the mean/median, standard deviation, and possible ceiling or floor effects of the different variables. The effect of the treatment on the primary outcome will be modeled using linear mixed models. *Post-hoc* comparisons will be carried out to test for differences in outcomes between the different time points, using Tukey's correction for multiple comparisons. In order to avoid statistical threats such as regression toward the mean, data will be analyzed as a whole, and not split up into groups based on the cognitive performances. The most recent version of IBM SPSS Statistics (IBM; Armonk, NY) and R (R Development Core Team; Vienna, Austria) will be used for the analyses.

Given the previous study conducted by Mosnier et al. (2015) it is hypothesized that, in general, the cognitive abilities of the older adults will increase at 6 and12 months after implantation. After this initial increase a gradual decrease in cognition is expected, similar to the age-related cognitive decline in normal hearing subjects. In other words, the authors expect the RBANS-H total percentile to increase during the first year after implantation, i.e., the hearing impaired participants will catch-up with the normal hearing norm group of the RBANS, and stabilize over the next nine years, i.e., the hearing impaired participants will show a cognitive decrease similar to the norm group.

### Data management

In order to enter and store the data in a secure, efficient, and clean manner, OpenClinica LLC is used. This is a software package for electronic data capture and data management developed for clinical research. This database is protected by a combination of user IDs and passwords and is only accessible to the principal investigators. Validation checks (e.g., range checks) for data values are programmed in order to minimize the number of mistakes while entering data. The information collected from this study is kept confidential. Individual information and results in the database are coded and only the researcher knows which code refers to which subject. The information is not shared with or given to anyone.

## Discussion

The primary aim of this prospective, longitudinal cohort study is to investigate the cognitive profile in older adults aged 55 or more with a profound hearing loss before and after cochlear implantation, both in the short and the long term. Secondly, due to the extensive audiological, cognitive, and qualitative examination, the current study also provides the opportunity to explore the relationship between the cognition, audiometric performances, quality of life and self-reliance in these patients. Before these research questions can be tackled, the feasibility and the validity of the RBANS-H to test hearing impaired older adults will be evaluated with regard to the following three questions: Are the written instructions clear to the participants? Do the modifications in the subtests *List Learning, Story Memory, Digit Span*, and *List Recognition* make it possible for the patients to fulfill these subtests? Is the RBANS-H sufficiently sensitive to detect changes in this population?

To the best of our knowledge, only one study conducted by Mosnier et al. (2015) investigated the effect of cochlear implantation on cognition in 94 subjects aged 65 or older. They used a test battery comprising six tests: Mini-Mental State Examination (MMSE) (Folstein et al., 1975; Derouesne et al., 1999; Lechevallier-Michel et al., 2004), 5-word test (Grober et al., 1988; Dubois et al., 2002; Cowppli-Bony et al., 2005), clock-drawing test (Solomon et al., 1998), verbal fluency test (Cardebat et al., 1990), d2 test of attention (Brickenkamp and Zillner, 1990), and trail making test (Tombaugh, 2004). By means of these tests, attention, memory, orientation, executive function, mental flexibility and fluency were assessed. These tests were performed preoperatively and at 6 and 12 months postoperatively. Mosnier et al. (2015) concluded that hearing rehabilitation through cochlear implantation results in improvements of cognitive abilities: more than 80% of the subjects with the poorest cognitive scores preoperatively improved their cognitive function significantly one year after implantation. In contrast, the cognitive capabilities of the majority of the subjects with normal scores preoperatively did not change significantly postoperatively. However, in 24% of these subjects a slight but significant decline in cognitive function was observed after the implantation.

The current study shares a comparable purpose as the study of Mosnier et al. (2015). Therefore, the study protocol of the current study is similar to the study protocol of Mosnier et al. (2015) with regard to the overall study design: both are prospective, longitudinal cohort studies concerning the cognitive functions of hearing impaired older adults with fixed test moments before implantation and at 6 and 12 months after implantation. Cognition, audiological performances and quality of life are investigated in both studies. However, the current study aims to investigate the cognitive trajectory not merely in the short term, but also in the long term, up to 10 years after implantation. Furthermore, in order to detect mild cognitive impairments at an early age, subjects of 55–65 years old are included as well. Finally, the major added value of the current study is that it does not make use of a composite test battery, but that it uses instead a comprehensive test, the RBANS-H, a cognitive assessment adjusted for use in hearing impaired subjects. This test makes it possible to calculate one total score of overall cognition, and also provides the opportunity to calculate the index scores per domain *Immediate Memory, Visuospatial/constructional, Language, Attention*, and *Delayed Memory*. The RBANS is sensitive to detect mild cognitive impairments in individuals aged 12–89 providing extended normative data per age category (12–13, 14–15, 16–19, 20–39, 40–49, 50–59, 60–69, 70–79, 80–89). The test effectuates the possibility to differentiate in a wide range from normal to moderately severe cognitive impairments. Another major advantage of the RBANS is that this test has a good sensitivity to change with 90 and 95% confidence intervals provided for the total score as well as each index score. The test is validated and available in English and Spanish, and easy to translate to other languages through the process of forward-backward translation. It takes around 30 min to administer the RBANS.

In contrast to the RBANS, the screening test Montreal Cognitive Assessment (MoCA) merely takes 10–15 min to administer and can be downloaded online for free (http://www.mocatest.org). However, screening tests such as MoCA are less sensitive and only differentiate between normal and abnormal cognitive function. In Table 4 a summary is given of the advantages and the limitations of the MoCA and the RBANS.

**Table 4:** **A schematic representation of the comparison between MoCA and RBANS**.

	**MoCA**	**RBANS**
Languages	>50 languages	English and Spanish
Public domain?	Yes	No
Duration	10–15 min	±30 min
Differentation in level of cognition	Normal *OR* mild cognitive impairment (screening)	Normal *TO* moderately severe dementia (scaled scores and percentiles with extensive normative data)
Index scores per cognitive domain	No	Yes (5)
Sensitivity to change	Limited	Good (+90 and 95% confidence intervals for the overall score and each index score)

Adding visual support to the subtests *List Learning, Story Memory, Digit Span*, and *List Recognition* was considered necessary to test the hearing impaired subjects in a reliable and identical manner before and after implantation and to ascertain that a possible improvement on the cognitive test cannot be attributed to the improved hearing with the CI (Phillips, 2016). According to the study of Delogu et al. (2009), the addition of visual stimulation in a verbal auditory memory task has no significant effect on the number of words repeated by the subjects. Since the auditory presentation is dominant in the case of verbal stimuli and there is no additional benefit from a bimodal (written and spoken) encoding of words, the modifications performed in this study are hypothesized to have little impact on the results. Nevertheless, these modifications may still have a small effect on the test scores and may invalidate the rigid use of the norms. By exploring the change in test and index scores, rather than the test and index scores *per se*, the effects of the modifications are reduced to the minimal.

In the proposed study, no control group is included. The discussion concerning the control group has to be situated in the background of increasing the level of evidence-based medicine for surgical procedures. Due to the absence of a control group, the effect of cochlear implantation on the cognition can, theoretically, not be separated from any change in cognition that occurs over time and no conclusion with regard to the causality of any observed changes can be rigorously taken. In order to answer the causality question whether cochlear implantation affects cognition and whether cochlear implantation can decelerate the age-related cognitive decline a different study design is required, such as a randomized clinical trial design. Obviously, due to ethical restrictions and the invasive nature of the intervention, it is impossible to randomly assign subjects, who all meet the criteria for reimbursement of cochlear implantation, to either the interventional or to the non-implanted control group.

Another option is a randomized delayed-start design, in which half of the participants are randomly assigned to either the immediate-intervention group or the delayed-intervention group. The Belgian national criteria for reimbursement of cochlear implantation are stricter than the internationally accepted minimal criteria for reimbursement. As a consequence, in the current study subjects with more severe hearing losses are recruited than would be the case in other countries. Even more postponing the implantation of a given participant would be unethical and would not be accepted by the patient, making it impossible to include the necessary number of subjects in a reasonable amount of time.

Furthermore, even without a random assignment of each participant to either the control group or the interventional group, the inclusion of a control group involves a number of problems which may either way bias the results. The subjects in the control group would ideally have the same audiological profile as the subjects in the interventional group but must not be implanted in the next 10 years. Since these subjects, like the subjects in the interventional group, do meet the criteria for reimbursement of cochlear implantation, the reason why they will not be implanted after all, may be also the reason why they should not be included in a control group, for instance because of a severe cognitive dysfunction or a complex health problem.

Therefore, the present study does not include a control group, but neither aims to point out whether cochlear implantation can decelerate the age-related cognitive decline or not. Instead, the study's purpose is to explore the cognitive profile of profoundly hearing impaired older adults before and after cochlear implantation using a comprehensive cognitive assessment and to investigate the relationship between cognition, audiometric performances, quality of life and self-reliance. Moreover, the current study may incite the design of more studies concerning cochlear implantation and cognition, such as studies assessing cognitive function in severely hearing impaired older adults who are not implanted and comparative simultaneous multicenter case-control studies taking advantage of the international differences between the criteria for reimbursement of cochlear implantation.

## Conclusion

The proposed exploratory study aims to give more insight into the cognitive profile of severely to profoundly hearing impaired older adults before and after cochlear implantation and into the relationship between their cognitive capabilities, audiometric performances, quality of life and self-reliance. The study may be a next step for future research investigating the relationship between hearing loss and cognition and the effects of cochlear implantation on this relationship, as to whether cognitive tests are important during the CI selection process and the rehabilitation and whether cochlear implantation could extend the period of independence in the older population.

## Dissemination

According to the Standard Protocol Items: Recommendations for Interventional Trials (SPIRIT) guidelines, the authors declare that a clinical article will be written on the primary and secondary outcomes of the study and will be disseminated regardless of the magnitude or direction of effect. The present trial is not industry initiated; therefore, there are no publication restrictions imposed by sponsors. In addition, a full study report, anonymized participant-level dataset and statistical code for generating the results will be made publicly available no later than 3 years after termination of the study for sharing purposes.

## Author contributions

AC, GM, AG, AH, VV, and PV conceived and designed the study. AC, GM, and PV drafted the manuscript, are coordinating the trial and are undertaking data collection and analysis. AG contributed to the quality of the manuscript and plays a role in data collection and analysis. AH made contributions to the manuscript and data collection. EF contributed to the manuscript and, more specifically, to the statistical analyses. VV critically revised the manuscript. All authors read and approved the final manuscript.

## Author information

AC is Master of Science in Audiology and a 2nd year PhD student at the Antwerp University Hospital/ University of Antwerp. GM is audiologist and a postdoctoral researcher at the Antwerp University Hospital/ University of Antwerp with special focus on cochlear implantation research. AG is audiologist and a postdoctoral researcher at the Antwerp University Hospital/ University of Antwerp with special focus on tinnitus research and lecturer at the University College Ghent. AH is the head audiologist at the Antwerp University Hospital. EF obtained a PhD in biochemistry at the University of Antwerp, is a postdoctoral researcher and professor in tenure at the University of Antwerp. VVR is head and neck surgeon and postdoctoral researcher at the Antwerp University Hospital and assistant professor at the University of Antwerp. PV is head of the Department of Otorhinolaryngology and Head and Neck Surgery at the Antwerp University Hospital and full professor in tenure at the University of Antwerp.

### Conflict of interest statement

The authors declare that the research was conducted in the absence of any commercial or financial relationships that could be construed as a potential conflict of interest. The Antwerp University Hospital is currently receiving a research grant from the company MED-EL, Innsbruck. The study was supported by a TOPBOF grant of the University of Antwerp.

## Abbreviations

CI: Cochlear Implant
dB HL: Decibel Hearing Level
dB SPL: Decibel Sound Pressure Level
dBnHL: Decibel normal Hearing Level
GCP: Good Clinical Practice
HADS: Hospital Anxiety and Depression Scale
HISQUI19: Hearing Implant Sound Quality Index-19
Hz: Hertz
LIST: Leuven Intelligibility Sentences Test
MMSE: Minimental State Examination
MoCA: Montreal Cognitive Assessment
MOM: Minimal Outcome Measurements
NCIQ: Nijmegen Cochlear Implant Questionnaire
NVA: Nederlandse Vereniging voor Audiologie
QoL: Quality of Life
RBANS: Repeatable Battery for the Assessment of Neuropsychological Status
RBANS-H: Repeatable Battery for the Assessment of Neuropsychological Status for Hearing impaired patients
SPIRIT: Standard Protocol Items: Recommendations for Interventional Trials
SRT: Speech Reception Threshold
SSQ12, Speech: Spatial and Qualities of Hearing scale-12
WAIS: Wechsler Adult Intelligence Scale.
