# Supplementary material for: The Repeatable Battery for the Assessment of Neuropsychological Status for Hearing Impaired Individuals (RBANS-H) before and after Cochlear Implantation: A Protocol for a Prospective, Longitudinal Cohort Study
Source: Front Neurosci. 2016 Nov 15;10:512. doi: 10.3389/fnins.2016.00512 (PMC5108794; doi:10.3389/fnins.2016.00512)
Supplement: Supplementary file 1 [file Image1.PDF]

Hearing Implant Sound Quality Index ($HSIQUI_{19}$)

1. Can you effortlessly distinguish between a male and a female voice?
2. When talking on the phone, can you effortlessly understand the voices of familiar people?
3. When listening to music, can you effortlessly distinguish whether one or multiple instruments are being played simultaneously?
4. When background noise is present, can you effortlessly participate in a conversation with friends or family members (e.g. at a party/ in a restaurant)?
5. Can you effortlessly hear noises such as falling keys, the beeping of the microwave or the purring of a cat?
6. Can you effortlessly distinguish single instruments in a familiar piece of music?
7. You are watching a movie on TV and music is playing in the background. Provided that the volume of the TV is loud enough, can you effortlessly understand the movie's text?
8. When talking on the phone, can you effortlessly understand the voices of unfamiliar people?
9. Can you effortlessly understand a speech/ lecture in a hall (e.g. lecture hall, church)?
10. Can you effortlessly distinguish between a female voice and a child's voice (6-10 years of age)?
11. At home when other family members are having a conversation and you are listening to the news on the radio, can you effortlessly under- stand the news?
12. Can you effortlessly understand the announcement in a bus terminal, a train station or an airport?
13. Can you effortlessly hear the ringing of the phone?
14. You are listening to friends or family members talking to each other in quiet surroundings. Can you effortlessly identify the talker?
15. You are seated on the back seat of a car and the driver in the front is talking to you. Can you effortlessly understand the driver?
16. Can you effortlessly allocate background noise to a specific sound source (e.g. toilet flushing or vacuum cleaner) using acoustic help only?
17. When other people in your close surrounding are having a conversation (e.g. talking to a salesperson, a bank clerk at the counter or a waiter in a busy restaurant), can you effortlessly talk to another person?
18. When background noise is present (e.g. in the office, printer, copier, air conditioning, fan, traffic noise, in busy restaurants, at parties, noisy children), can you effortlessly participate in a conversation with multiple people?
19. When multiple people are talking simultaneously, can you effortlessly follow discussions of friends and family members?
