# Supplementary material for: The Repeatable Battery for the Assessment of Neuropsychological Status for Hearing Impaired Individuals (RBANS-H) before and after Cochlear Implantation: A Protocol for a Prospective, Longitudinal Cohort Study
Source: Front Neurosci. 2016 Nov 15;10:512. doi: 10.3389/fnins.2016.00512 (PMC5108794; doi:10.3389/fnins.2016.00512)
Supplement: Supplementary file 2 [file Image2.PDF]

Nijmegen Cochlear Implant Questionnaire (NCIQ)

1. Can you hear background noises (toilet flushing, vacuum cleaner)?
2. Does your hearing impairment present a serious obstacle in your contact with persons with normal hearing?
3. Are you able to whisper if you have to?
4. Do you feel at ease in company despite your hearing impairment?
5. Can you hold a conversation in a quiet environment (with or without lip-reading) with one person?
6. Does your hearing impairment present a serious problem during your work or studies?
7. Can you hear the footsteps of other persons in your house (eg, in the hall or on the stairs)?
8. Does your hearing impairment present a serious problem in your contact with deaf persons?
9. Are you able to shout if you need to?
10. Does it bother you that you are hard of hearing?
11. Are you able to hold a conversation with 2 or more persons in a quiet environment (with or without lip reading)?
12. Does your hearing impairment present a serious problem in traffic?
13. Can you hear your own telephone or doorbell ringing?
14. Does your hearing impairment present a serious problem when you are with a group of persons (hobbies, sport, holidays)?
15. Are you able to make yourself understood to strangers without using hand gestures?
16. Do you become irritated if you cannot follow a conversation?
17. When you are in a busy shop, can you understand the shop assistant?
18. Does your hearing impairment present a serious problem during leisure-time activities?
19. Can you hear (not feel) the front door slam when you are busy at home?
20. Does your hearing impairment present a serious problem in your contact with the persons you live with (family/ partner)?
21. Are you able to adapt your voice to different situations (noisy environment, quiet environment)?
22. Do you avoid speaking to strangers?
23. Are you able to enjoy music?
24. Does your hearing impairment present a serious problem for functioning in the home?
25. Are you able to hear cars approaching in traffic?
26. Are you left aside in company because of your hearing impairment?
27. Can strangers hear from your voice that you are deaf or hearing-impaired?
28. Do you ask other persons to speak more loudly or clearly if they are speaking too softly or unclearly?
29. Are you able to recognize certain melodies in music?
30. Does your hearing impairment present a serious problem when you are shopping?
31. Can you hear soft noises (key falling, microwave beeping)?
32. Do you go places where your hearing impairment might present a serious handicap?

33. Can you make yourself understood to acquaintances without using hand gestures?
34. Do you feel anxious when talking to strangers?
35. Are you able to recognize certain rhythms in music?
36. Does your hearing impairment present a serious problem when watching television?
37. Can you hear (not feel) someone approaching you from behind?
38. Does your hearing impairment present a serious hindrance in your contact with persons who live in your neighborhood?
39. How often does it annoy you that persons can hear from your voice/speech that you have a hearing problem?
40. Can you understand strangers without lip-reading?
41. Does your hearing impairment present a serious problem at parties (eg, birthday)?
42. Can you hear (not necessarily understand) persons talking on the radio?
43. Does your hearing impairment present a serious problem when you are with friends?
44. Can you make contact easily with other persons despiteyour hearing problem?
45. Can you hear the difference between a man's voice, a woman's voice, and a child's voice?
46. Does your hearing impairment present a serious problem when dealing with formal matters (insurance, solicitor, municipal office)?
47. Can you hear when someone calls you?
48. Does your hearing impairment present a serious problem in your contacts with family members?
49. Are there situations in which you would feel happier if you were not hearing-impaired?
50. Do you feel it tiring to listen (with or without lip-reading)?
51. Does your hearing impairment present a serious problem when you go out or go on trips?
52. Can you hear voices from another room (eg, children playing, baby crying)?
53. When you are in a group, do you feel that your hearing impairment keeps persons from taking you seriously?
54. Does your hearing impairment undermine your self-confidence?
55. Does your hearing impairment prevent you from sticking up for yourself (at work, in relationships)?
56. Are you able to make your voice sound angry, friendly, or sad?
57. Can you control the pitch of your voice (high, low)?
58. Can you control the volume of your voice?
59. Can you make your voice sound "natural" (so that is does not sound like a deaf person's voice)?
60. Are you able to hold a simple telephone conversation?
