# Supplementary material for: The Repeatable Battery for the Assessment of Neuropsychological Status for Hearing Impaired Individuals (RBANS-H) before and after Cochlear Implantation: A Protocol for a Prospective, Longitudinal Cohort Study
Source: Front Neurosci. 2016 Nov 15;10:512. doi: 10.3389/fnins.2016.00512 (PMC5108794; doi:10.3389/fnins.2016.00512)
Supplement: Supplementary file 3 [file Image3.PDF]

Speech, Spatial and Qualities of Hearing scale (SSQ12)

1. You are talking with one other person and there is a TV on in the same room. Without turning the TV down, can you follow what the person you're talking to says?
2. You are listening to someone talking to you, while at the same time trying to follow the news on TV. Can you follow what both people are saying?
3. You are in a conversation with one person in a room where there are many other people talking. Can you follow what the person you are talking to is saying?
4. You are in a group of about five people in a busy restaurant. You can see everyone else in the group. Can you follow the conversation?
5. You are with a group and the conversation switches from one person to another. Can you easily follow the conversation without missing the start of what each new speaker is saying?
6. You are outside. A dog barks loudly. Can you tell immediately where it is, without having to look?
7. Can you tell how far away a bus or a truck is, from the sound?
8. Can you tell from the sound whether a bus or truck is coming towards you or going away?
9. When you hear more than one sound at a time, do you have the impression that it seems like a single jumbled sound?
10. When you listen to music, can you make out which instruments are playing?
11. Do everyday sounds that you can hear easily seem clear to you (not blurred)?
12. Do you have to concentrate very much when listening to someone or something?
