# Supplementary material for: The Repeatable Battery for the Assessment of Neuropsychological Status for Hearing Impaired Individuals (RBANS-H) before and after Cochlear Implantation: A Protocol for a Prospective, Longitudinal Cohort Study
Source: Front Neurosci. 2016 Nov 15;10:512. doi: 10.3389/fnins.2016.00512 (PMC5108794; doi:10.3389/fnins.2016.00512)
Supplement: Supplementary file 4 [file Image4.pdf]

Hospital Anxiety and Depression Scale (HADS)

A (1) I feel tense or wound up:
- Most of the time (3)
- A lot of times (2)
- From time to time (1)
- Not at all (0)

D (2) I still enjoy the things I used to:
- Definitely as much (0)
- Not quite so much (1)
- Only a little (2)
- Hardly at all (3)

A (3) I get a sort of frightened feeling as if something awful is about to happen:
- Very definitely and quite badly (3)
- Yes, but not too badly (2)
- A little, but it doesn't worry me (1)
- Not at all (0)

D (4) I can laugh and see the funny side of things:
- As much as I always could (0)
- Not quite as much now (1)
- Definitely not so much now (2)
- Not at all (3)

A (5) Worrying thoughts go through my mind:
- Most of the time (3)
- A lot of times (2)
- From time to time (1)
- Only occasionally (0)

D (6) I feel cheerful:
- Most of the time (0)
- Usually (1)
- Not often (2)
- Not at all (3)

A (7) I can seat at ease and feel relaxed:
- Definitely (0)
- Usually (1)
- Not often (2)
- Not at all (3)

D (8) I feel as I am slowed down:
- Nearly all the time (3)

Very often (2)
From time to time (1)
Not at all (0)

A (9) I get a sort of frightened feeling like butterflies in the stomach:
Not at all (0)
From time to time (1)
Quite often (2)
Very often (3)

D (10) I have lost interest in my appearance:
Definitely (3)
I don’t take so much care as I should (2)
I may not take quite as much care (1)
I take just as much care as ever (0)

A (11) I feel restless, as if I had to be on the move:
Very much indeed (3)
Quite a lot (2)
Not very much (1)
Not at all (0)

D (12) I look forward with enjoyment to things:
As much as I ever did (0)
A little less than I used to (1)
Definitely less than I used to (2)
Hardly at all (3)

A (13) I get a sudden feeling of panic:
Very often indeed (3)
Quite often (2)
From time to time (1)
Not at all (0)

D (14) I can enjoy a good TV or radio program or book:
Often (0)
Sometimes (1)
Not often (2)
Hardly at all (3)
